# Diphtheria Antitoxin

**Published:** 1902-09

**Authors:** 


### DIPHTHERIA ANTITOXIN.

---

An able paper on "Serum Therapy," read by Dr. H. N. Graves, of Georgetown [Dr. Graves's pàper will appear in next issue.—Ed.], at a recent meeting of the West Texas Medical Association developed an interesting, and, withal, a spirited discussion as to the value of antitoxin in the treatment of diphtheria. The discussion was, numerically at least, one-sided, for all but two of the many speakers agreed with Dr. Graves that antitoxin has brought about an enormous decrease in the death rate, and that since its introduction all other treatment has been rendered unnecessary. Many cases of marvelous cures were reported. For example, one of the speakers, merely as a routine measure, had injected three thousand units of diphtheria antitoxin into a small child whom he considered to be already beyond human aid, and was astonished on the following morning to find the patient, not only alive, but sitting up in bed, apparently entirely recovered. Other cases not less wonderful were discussed. On the other side, Dr. C. E. R. King, of San Antonio, characterized the entire doctrine of serum therapy as "absurd," and expressed his belief that the good results, if any, that followed the injection of anti-diphtheritic serum are due to the carbolic acid used in it as a preservative.

While not wishing to endorse the contention of the minority side in the debate, we cannot forbear to call attention to the fact that the much talked of statistics referred to by the majority side are, to say the least, misleading. In the first place it is exceedingly difficult to determine a rise or fall in the death rate in this or any other similar disease from a mere study of the records during given periods. Epidemics vary greatly in virulence, one with a mortality of ten per cent. being frequently followed by another with a mortality of fifty or sixty per cent., and *vice versa*. In 1802, during a widespread epidemic in some of the larger cities of Continental Europe, the death rate is said to have been as low as two per cent. The perfection of the microscope has given rise to another source of error. Since that instrument has come into use many cases with practically no clinical symptoms are now diagnosed as diphtheria and reported as such. Their inclusion, of course, materially lowers the death rate as it appears on the records.

A mere comparative study of the mortality statistics, together with the very limited personal experience that most practitioners have had, is not sufficient evidence upon which to form an opinion (except tentatively) as to the value of the antitoxin treatment. However, we would certainly not advise against its use. Since it does no harm and does not interfere with other means of treatment, by its use we have everything to gain and nothing to lose. Under the circumstances it is clearly every man's duty to make use of it in every case. However, the unqualified endorsement and enthusiastic praise that many men give to it do not seem justified at the present time. R.

